# Adverse Childhood Experiences and Sarcopenia in Later Life: Baseline Data from the Canadian Longitudinal Study on Aging

**DOI:** 10.3390/geriatrics10040111

**Published:** 2025-08-15

**Authors:** Menelaos M. Dimitriadis, Kitty J. E. Kokkeler, Emiel O. Hoogendijk, Radboud M. Marijnissen, Ivan Aprahamian, Hans W. Jeuring, Richard C. Oude Voshaar

**Affiliations:** 1 Department of Psychiatry, University of Groningen, University Medical Center Groningen, Hanzeplein 1, 9713 GZ Groningen, The Netherlands; 2 Department of Old Age Psychiatry, ProPersona, Wolfheze 2, 6874 BE Wolfheze, The Netherlands; 3 Department of Epidemiology & Data Science, Amsterdam Public Health Research Institute, Amsterdam UMC—Location VU University Medical Center, Van der Boechorststraat 7, 1081 BT Amsterdam, The Netherlands; 4 Geriatrics Division & GIMMA Translational Research Group, Jundiaí Medical School, Jundiaí 13202-550, São Paulo, Brazil

**Keywords:** adverse childhood experiences, ACE, sarcopenia, frailty, aged, aging

## Abstract

Backgrounds: Adverse Childhood Experiences (ACEs) are linked to early and long-lasting mental health issues and somatic multimorbidity. Emerging evidence suggests ACEs may also accelerate physical frailty in old age. This study examines the association between ACEs and sarcopenia, an ageing-related disease and core component of frailty. Methods: Baseline data from the Canadian Longitudinal Study on Aging (CLSA), including 25,327 participants aged 45–85 years (50.3% female sex) were analyzed. Sarcopenia was defined using the revised European Working Group of Sarcopenia in Older People (EWGSOP2) guidelines. ACE were assessed via the Childhood Experiences of Violence Questionnaire and the National Longitudinal Study of Adolescent to Adult Health Wave III questionnaire, covering eight ACE categories. Multiple logistic regression models examined the association between the number of ACE count and sarcopenia, which were adjusted for age, sex, education, income, and ethnicity. Results: Given a significant interaction between age and ACE (*p* < 0.01), analyses were stratified into four age groups (45–54, 55–64, 65–74, and 75–85 years). A significant association only emerged in the oldest group (75–85 years; OR = 0.93 [95% CI: 0.86–1.00], *p* = 0.043), but this result was in the opposite direction we hypothesized. Sensitivity analyses confirmed findings across different operationalisations of ACE and sarcopenia. Conclusions: Higher ACE exposure was not associated with sarcopenia in middle aged and older adults. The unexpected protective association in the oldest-old subgroup may reflect survival bias. Age-stratified longitudinal studies are needed to clarify this relationship.

## 1. Introduction

Sarcopenia is characterized by the progressive loss of muscle mass, strength, and function [1], affecting 10 to 27% of the population over 60 years of age [1,2]. Initially considered a normal consequence of aging, sarcopenia is now recognized as a disease under the Clinical Modification of the 10th International Classification of Diseases (ICD-10-CM, code: M62.84) [3]. Growing evidence indicates that sarcopenia is a multifactorial condition associated with numerous adverse health effects [4], including increased risk of falls and fractures, impaired activities of daily living, cardiovascular and respiratory diseases, cognitive decline, reduced quality of life, loss of independence, and mortality [1]. Sarcopenia also imposes substantial societal costs due to its contribution to (prolonged) hospitalization [1]. Identifying determinants of sarcopenia is therefore crucial for developing preventive strategies. In this study, we focus on adverse childhood experiences (ACEs) as a potential risk factor.

While initial studies on ACEs emphasized their link with psychiatric disorders, accumulating evidence also suggests associations with somatic diseases in midlife [5,6]. Recent studies have identified ACEs as independent risk factors for frailty and its progression in later life [7,8,9]. As sarcopenia constitutes a key biological substrate of biomedical frailty, especially of the frailty phenotype [4,10,11], one may hypothesize that ACEs are also associated with sarcopenia. To our knowledge, this association has only been examined in a middle-aged and older Chinese population, where a significant association was observed [12]. These initial findings challenge the view of sarcopenia as exclusively a problem of old age and suggest that frailty prevention efforts should begin earlier in life.

ACEs, defined as traumatic events occurring during childhood, have been consistently linked to the onset of psychiatric disorders [13]. While the link to physical health outcomes has often been hypothesized to be mediated by mental health problems [13], alternative direct pathways have also been proposed, involving pathophysiological mechanisms (e.g., low-grade inflammation and hypercortisolism), and unhealthy behavior (e.g., low physical activity). Moreover, these pathways are not mutually exclusive, and components of most pathways, as well as the onset of multimorbidity in midlife, have also been associated with sarcopenia [14,15]. These potential mechanisms were not considered in the only previous study addressing the association between ACEs and sarcopenia [12].

We hypothesize that individuals who have experienced ACEs have a higher prevalence of sarcopenia in middle and older adulthood compared to those without ACEs. Secondly, we explore whether the potential association between ACEs and sarcopenia may be partly explained by any of the abovementioned pathways. Confirming this hypothesis could offer novel insights into the mechanisms by which ACEs exert long-term health effects and inform early-life prevention strategies.

## 2. Materials and Methods

### 2.1. Sampling and Design

The study was embedded in the Canadian Longitudinal Study on Aging (CLSA) cohort, including 51,338 participants aged 45–85 years from 10 Canadian provinces. People who were not able to participate due to physical or cognitive reasons or participants that were living in institutions were excluded from the CLSA. All participants of the CLSA provided written informed consent for their participation. More information about the CLSA can be found elsewhere [16,17]. The Ethical Committee (METC) of the University Medical Center Groningen provided ethics approval for use of the deidentified data (METc2022/069).

For the present cross-sectional study, we selected the comprehensive cohort of the CLSA that received in-depth physical assessments, including blood collection and assessment of sarcopenia. This cohort includes 30,097 participants who were randomly selected from within 25–50 km radius from the data collection sites of CLSA in seven Canadian provinces. Since ACEs were assessed during the first follow-up wave of CLSA, those who dropped out before the ACE assessment were also excluded for the present study. A total of 25,327 out of the 30,097 participants, who had no missing data on either ACE or sarcopenia, were included in this study.

### 2.2. Primary Outcome (Sarcopenia)

The presence of sarcopenia (yes/no) was constructed according to the revised European Working Group on Sarcopenia in Older People (EWGSOP2) criteria [1]. Sarcopenia was defined as the combination of (1) decreased muscle strength and (2) decreased muscle quantity. If only one of the two criteria was met or neither was met, then the specific participant was considered to not have sarcopenia.

Decreased muscle strength was based on either hand grip strength or the chair rise test. Participants qualified for decreased muscle strength if they scored below the cutoff in 1 (or both) of the tests. Hand grip strength was measured using a Wireless Grip Dynamometer (JTech Tracker Freedom, Midvale, UT, USA). The highest score was used from three repetitive attempts using the participant’s dominant hand. The chair rise test involved participants rising to a fully standing position from a seated position five times as quickly as possible, with time measured in seconds. Sex-specific cutoff values for decreased muscle strength were used according to the EWGSOP2 criteria [17].

Muscle quantity was defined as lean muscle mass and measured by a Dual X-ray absorptiometry (DXA) scan adjusted for height. The DXA machine was calibrated daily using a spine phantom, weekly using a whole-body step phantom, and yearly using a gold standard phantom [17].

### 2.3. Independent Variable (ACE)

Adverse childhood experiences were measured using the short form of the Childhood Experiences of Violence Questionnaire (CEVQ) and the National Longitudinal Study of Adolescent to Adult Health Wave III questionnaire [18,19,20], as described previously in detail [21]. All items referred to exposure before the age of 16 years. Frequency of exposure to childhood physical abuse, sexual abuse, emotional abuse, neglect, and intimate partner violence were assessed on an ordinal scale (never, 1–2 times, 3–5 times, 6–10 times, or more than 10 times) and subsequently dichotomized as presence or absence of exposure based on the CEVQ instructions. Other forms of ACE, including “parental divorce or separation,” “parental death”, or “living with a family member with mental health problems”, were assessed dichotomously. A cumulative ACE score was created by summing the number of individual ACEs that participants had experienced and ranged from 0 to 8.

### 2.4. Covariates

As potential confounders, we included age, sex, level of education, household income, and Caucasian ethnicity (yes/no), which are all associated with either ACEs or sarcopenia and are unlikely a direct consequence of ACEs [22,23,24]. Educational level was categorized in four levels and defined as lower (less than secondary school graduation), middle, bachelor level, or master level and above. Income category was recategorized from five levels into three levels, i.e., less than CAD 50,000 annually, CAD 50,000 to CAD 100,000 annually, or more than CAD 100,000 annually.

Variables that may be a consequence of ACE, and thus may lie on the pathway from ACE to sarcopenia, were considered as potential mediators in the exploratory analyses (see below). We evaluated mental health, somatic health, behavioral, and physiological pathways.

Regarding mental health, we included lifetime depression, which was based on the self-report question “Did a doctor ever diagnose you with having a depression?”.

Regarding somatic health, we included the total number of registered diseases for each participant, based on self-reported data supplemented, where necessary by additional evaluations such as imaging (e.g., X-rays), physical assessments (e.g., blood pressure), and laboratory measures (e.g., glucose), as previously described [25].

Regarding behavioral pathways, we included physical activity, as measured by using Physical Activity Scale for the Elderly (PASE) questionnaire [26].

Regarding physiological variables, we included C-Reactive Protein (CRP), as was measured by blood tests performed at baseline measurement [16,17].

### 2.5. Statistical Analyses

Descriptive statistics (Chi^2^-test or Student’s *t*-tests) were used to compare participants with and without sarcopenia in our study sample. To study the association between ACE and sarcopenia, multiple multinominal logistic regression analyses were performed with sarcopenia as the dichotomous dependent variable and the number of ACEs as the independent variable. In each step, we first checked for interaction effects between ACE and each covariate (confounder or explanatory variable) in their association with sarcopenia and proceeded to run stratified analyses when a significant interaction was found.

In accordance with a previous paper in CLSA [7], we performed sensitivity analyses by constructing 3 subgroups of participants that have experienced 1 ACE, 2 ACEs, or 3+ ACEs to be compared with participants with no ACE. All analyses were adjusted for confounders only (age, sex, ethnicity, level of education, income).

Subsequently, we evaluated whether lifetime depression, number of somatic comorbidities, physical activities, and CRP were mediators in the hypothesized associations. Mediation effects were tested for each potential mediator in separate models by using bootstrapping methods using the SPSS PROCESS macro version 4.3.1 developed by Preacher and Hayes [27].

All analyses have been conducted in IBM SPSS Statistics, version 28 (Armonk, NY, USA). *p*-values < 0.05 were considered statistically significant.

## 3. Results

### 3.1. Study Population

A total of 1840/25,327 (7.3%) of the population met the criteria for sarcopenia. Table 1 shows the baseline characteristics of our study sample stratified by the presence of sarcopenia. The participants with sarcopenia were older, more often female, less highly educated, were more often of a non-Caucasian ethnicity, had less ACEs, were more often diagnosed with depression during their lifetime, had more somatic diseases, and were less physically active than their counterparts with no sarcopenia.

**Figure 1:**
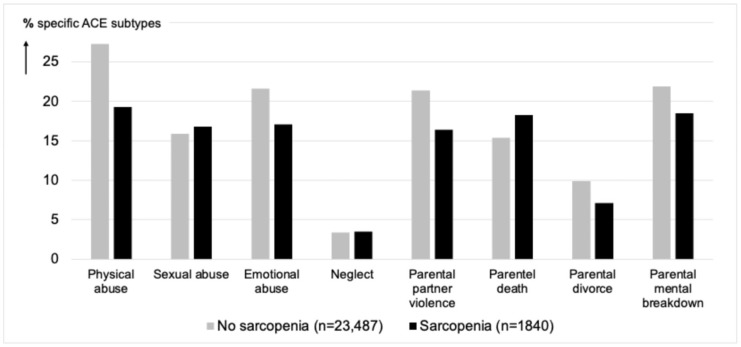
Prevalence (%) of adverse childhood experiences (stratified by sarcopenia).

The prevalence of sarcopenia was significantly higher among women (10.1%) compared to men (4.4%) (X^2^ = 300.5, df = 1, *p* < 0.001) and increased with age (45–54 years: 2.8%, 55–64 years: 4.6%, 65–74 years: 8.6%, and 75+: 18.6%; X^2^ = 1058.6; df = 3, *p* < 0.001). A total of 14745/23487 (62.8%) of the participants that were not sarcopenic had experienced at least one ACE, and 1071/1840 (58.2%) of the participants with sarcopenia (X^2^ = 15.2, df = 1, *p* < 0.001) experience at least one ACE. Figure 1 presents the prevalence of the specific subtypes of ACE assessed stratified by the presence of sarcopenia.

### 3.2. Association Between ACE and Sarcopenia

Checking the interaction between ACE and any of the covariates with sarcopenia as the dependent variable, we found only a significant the interaction with age (*p* = 0.008). We therefore stratified our analyses into four different age groups (45–54, 55–64, 65–74, and 75+ years).

Table 2 demonstrates the association between the number of ACEs and have sarcopenia across all four age groups against having no ACE. Only among participants aged 75 years or older did we find that people with a higher number of ACEs had significantly lower odds of having sarcopenia. These findings were confirmed by the sensitivity analyses analyzing the impact of having 1, 2, or ≥3 ACEs on having sarcopenia, showing that having two, three, or more ACEs among participants aged 75+ had significantly lower odds of having sarcopenia.

No associations were found between a higher number of ACEs and sarcopenia, so mediation analyses were not conducted.

### 3.3. Sensitivity Analyses

Considering these unexpected findings, we post hoc performed two additional sensitivity analyses to examine whether negative results could be explained by methodological choices made regarding the operationalization of either ACE or sarcopenia.

First, we examined whether a specific type of ACE might be associated with sarcopenia. As shown in Table 3, we found no significant associations between any specific type of ACE and sarcopenia (using logistic regression adjusted for age, sex, level of education, income, and ethnicity) in the three youngest age groups, while the overall finding in those aged 75 years and older seems to be driven by physical abuse and witnessing a parental divorce.

Secondly, we repeated all logistic regression analyses excluding participants scoring positive on only one of the two criteria for sarcopenia. These sensitivity analyses revealed no significant associations at all (see Table 4).

## 4. Discussion

Contrary to our hypothesis, we found no association between a higher number of ACEs and sarcopenia among middle-aged and older adults. In contrast, among the oldest -old subgroup (75+) we even found a negative association with ACE associated with a lower prevalence of sarcopenia. Naturally, our finding must be interpreted with some caution, as the relationship between ACE and sarcopenia has hardly been examined before. On the other hand, by using a well-designed large-population-based Canadian cohort study (CLSA), the finding is most likely reliable. Below, we discuss reasons why ACE might not be associated with sarcopenia.

A recent umbrella review on the long-term consequences of ACEs found substantial evidence for the association with mental health disorders and psychosocial sequelae, while studies on somatic health indices were methodologically flawed [13]. Furthermore, as the association between ACE and adverse somatic health effects has been less often examined, the association may also be overestimated by publication bias of positive findings only. On the other hand, the negative finding may be explained by selection bias, as people with the worst physical and mental health have been excluded in the CLSA. If true, one may hypothesize that the counterintuitive finding in the oldest-old subgroup is explained by bias due to survival of the fittest: ‘only those resilient for the detrimental effects of ACE will survive into old age’.

Nonetheless, our negative findings may still be compatible with a true association between ACE and frailty. First, frailty is not a unidimensional construct. The frailty index, one of the most dominant frailty models, captures multiple health deficits across several domains, including mental health. Although sarcopenia is a core component of the Frailty Phenotype, another dominant frailty model, the Frailty Phenotype is considered multidimensional, encompassing a performance-based and a vitality-based dimension, with the latter closely linked to psychosocial functioning [28]. Given this, it is plausible that the association between ACE and frailty is primarily driven by psychosocial pathways or mental health factors rather than physical deterioration alone. Supporting this view, a recent study reported partial mediation by lifestyle factors in the relationship between ACE and frailty [29]. Higher risk of apathy, loneliness, depression, substance abuse, and chronic inflammation could be (part of) the mediating pathway underlying ACE and frailty in later life [5,14,30], also driving the deficit accumulation model which substantiates the frailty index [7,9].

Finally, the effects of ACE across the whole biopsychosocial domain have hardly been examined longitudinally. Longitudinal analyses would be highly relevant to confirm or refute the potentially protective effect of ACE on sarcopenia in the oldest old. If our results are driven by survival bias, longitudinal studies may still reveal a positive association between ACE and onset of sarcopenia in this age group. Longitudinal studies in later life would also be important to examine whether ACE only exerts negative health effects early in life that are not restored during aging or whether it still exerts ongoing effects in middle and older age. Since sarcopenia is an age-related disease affecting the oldest old, with low prevalence rates in younger age groups; one may also hypothesize that the effect of ACE on sarcopenia only affects the oldest old. In line with this reasoning, ACEs were associated with the progression of frailty over time in the oldest old (70+) but not among middle-aged and older persons (58–69 years) in the Longitudinal Aging Study Amsterdam (LASA) [9]. Therefore, it may still be relevant to examine the association between ACE and sarcopenia longitudinally, as well as the impact of ACE on other somatic and mental health indices like cardiac disease, depression, and frailty [7,31].

### 4.1. Methodological Considerations

An important strength of this study was the utilization of a large and representative cohort, which conferred substantial statistical power to our analyses. The extensive dataset allowed us to investigate our research questions with a high degree of precision, resulting in robust findings in subgroups (by age) as well as sensitivity analyses.

A notable limitation was the retrospective measurement of adverse childhood experiences (ACEs). We acknowledge that retrospective data collection may introduce recall bias and memory inaccuracies, potentially affecting the accuracy and reliability of ACE information provided by participants. This concern has been addressed previously, but all studies concluded that memory bias due to retrospective recording of ACE was acceptable [7,8,21] and not related to mild cognitive impairment or cognitive performance [32]. Additionally, the relatively high prevalence of ACEs in the CLSA study sample (60% reporting at least one ACE) raises questions about the significance of that risk factor. However, it is important to note that when we conducted the analyses for the subgroup of individuals who had experienced three or more ACE, with a prevalence of 17%, no significant results were found either.

Secondly, we did not assess all potential mediators of the association between ACE and sarcopenia. For instance, dietary habits and substance use were not taken into account. Moreover, the study also lacked data on disease severity (for which polypharmacy might serve as a proxy) and did not differentiate between diseases based on their presumed strength of association with sarcopenia. However, given the negative findings, these limitations are unlikely to have substantially influenced our results.

### 4.2. Conclusions

The absence of an association between a higher number of ACEs and sarcopenia in this study suggests that sarcopenia may not play a role in the pathway linking ACE to adverse somatic health outcomes, such as frailty. However, to obtain more robust evidence on the long-term impact of ACE on health in the latest stages of life, replication studies—particularly longitudinal studies in the oldest age groups—are needed. These studies can account for potential survival bias and should consider possible mediating mechanisms.

## Figures and Tables

**Table 1:** Baseline characteristics stratified for the presence of sarcopenia (complete cases).

		Presence of Sarcopenia		
Characteristics		No (*n* = 23,487)	Yes (*n* = 1840)	Statistics
*Socio-demographics:*				
Age, years	mean (SD)	61.8 (9.8)	69.5 (10.2)	t = −32.5, df = 25,325, *p* < 0.001
Female sex	*n* (%)	11,462 (48.8)	1284 (69.8)	Chi^2^ = 300.5, df = 1, *p* < 0.001
Level of education, years				Chi^2^ = 146.4, df = 4, *p* < 0.001
◦ Low	*n* (%)	4125 (17.6)	366 (19.9)	
◦ Middle	*n* (%)	5001 (21.3)	417 (22.7)	
◦ Bachelor level	*n* (%)	5812 (24.7)	350 (19.0)	
◦ Master level	*n* (%)	5425 (23.1)	308 (16.7)	
◦ Missing	*n* (%)	3124 (13.3)	308 (21.7)	
Household income				Chi^2^ = 518.6, df = 3, *p* < 0.001
◦ Low	*n* (%)	5302 (22.6)	759 (41.3)	
◦ Middle	*n* (%)	7889 (33.6)	599 (32.6)	
◦ High	*n* (%)	9002 (38.3)	305 (16.6)	
◦ Missing	*n* (%)	1294 (5.5)	177 (9.6)	
Caucasian ethnicity	*n* (%)	22,569 (96.1)	1738 (94.5)	Chi^2^ = 11.8, df = 1, *p* < 0.001
*Independent variables:*				
Number of all different ACE	mean (SD)	1.4 (1.5)	1.2 (1.4)	t = 5.4, df = 25,325, *p* < 0.001
Number of ACE categorised:				Chi^2^ = 35.7, df = 3, *p* < 0.001
◦ No ACE	*n* (%)	8742 (39.2)	769 (43.6)	
◦ One ACE	*n* (%)	6425 (28.8)	551 (31.2)	
◦ Two ACEs	*n* (%)	3592 (16.1)	230 (13.0)	
◦ Three or more ACEs	*n* (%)	3540 (15.9)	214 (12.1)	
*Potential mediators:*				
Lifetime depression, lifetime	*n* (%)	3683 (15.7)	324 (17.7)	Chi^2^ = 4.7, df = 1, *p* = 0.031
Number of somatic diseases	mean (SD)	2.1 (1.8)	2.8 (2.1)	T = −15.7, df = 23574, *p* < 0.001
Physical activity (MET minutes)	mean (SD)	379.9 (98.4)	341.0 (91.8)	t = 16.4, df = 25,325, *p* < 0.001
hsCRP (mg/L)	mean (SD)	2.4 (4.7)	2.7 (6.3)	t = −2.1, df = 23,037, *p* = 0.102

Abbreviations: ACE: adverse childhood experience; SD: standard deviation.

**Table 2:** Association between adverse childhood experiences (ACEs) and sarcopenia using logistic regression analyses stratified by age and adjusted for potential confounders *.

	45–54 Years Old (*n* = 6714)			55–64 Years Old (*n* = 8560)			65–74 Years Old (*n* = 6123)			75+ Years Old (*n* = 3930)		
	OR	[95% CI]	*p*	OR	[95% CI]	*p*	OR	[95% CI]	*p*	OR	[95% CI]	*p*
*Main model*												
Experienced ACE	0.99	[0.90–1.07]	0.737	0.99	[0.93–1.06]	0.791	0.97	[0.91–1.04]	0.419	0.93	[0.86–1.00]	0.043
*Sensitivity analysis:*												
No ACE	REF		0.030	REF		0.481	REF		0.355	REF		0.032
One ACE	1.05	[0.72–1.53]	0.819	0.95	[0.73–1.25]	0.721	1.22	[0.98–1.51]	0.073	1.00	[0.83–1.21]	0.991
Two ACEs	0.45	[0.26–0.80]	0.006	1.14	[0.85–1.53]	0.391	1.07	[0.80–1.42]	0.653	0.72	[0.54–0.95]	0.021
Three or more ACEs	0.54	[0.54–1.28]	0.407	0.87	[0.63–1.19]	0.381	1.08	[0.81–1.45]	0.597	0.72	[0.52–1.00]	0.052

* Included covariates are age, sex, level of education, ethnicity, and household income.

**Table 3:** Associations between specific ACE types and sarcopenia by logistic regression analyses (adjusted for age, sex, level of education, ethnicity, and household income).

	45–54 Years			55–64 Years			65–74 Years			75+ Years		
ACE Subtypes	OR	[95% CI]	*p*	OR	[95% CI]	*p*	OR	[95% CI]	*p*	OR	[95% CI]	*p*
Physical abuse	1.00	[0.77–1.30]	0.986	1.00	[0.77–1.30]	0.986	0.82	[0.64–1.06]	0.130	0.63	[0.49–0.82]	<0.001
Sexual abuse	1.06	[0.82–1.38]	0.641	1.06	[0.82–1.38]	0.641	0.91	[0.70–1.17]	0.451	1.24	[0.96–1.61]	0.106
Emotional abuse	1.06	[0.80–1.42]	0.679	1.06	[0.80–1.42]	0.679	0.99	[0.75–1.32]	0.961	1.04	[0.76–1.41]	0.809
Neglect	0.91	[0.54–1.53]	0.708	0.91	[0.54–1.53]	0.708	1.12	[0.67–1.88]	0.664	1.11	[0.65–1.91]	0.702
Intimate partner violence	0.92	[0.69–1.23]	0.586	0.92	[0.69–1.23]	0.586	0.93	[0.70–1.23]	0.606	0.83	[0.60–1.14]	0.240
Witnessed parental death	0.87	[0.65–1.17]	0.353	0.87	[0.65–1.17]	0.353	1.12	[0.88–1.41]	0.366	1.13	[0.92–1.39]	0.231
Witnessed parental divorce	1.11	[0.78–1.59]	0.553	1.11	[0.78–1.59]	0.553	0.82	[0.55–1.23]	0.343	0.64	[0.42–0.96]	0.032
Witnessed parental mental breakdown	0.95	[0.74–1.22]	0.947	0.95	[0.74–1.22]	0.673	1.25	[0.99–1.59]	0.061	1.08	[0.83–1.40]	0.587

Abbreviations: ACE: adverse childhood experience; OR: odds ratio; CI: confidence interval.

**Table 4:** Association between adverse childhood experience (ACE) and meeting (at least) one component of sarcopenia using logistic regression analyses stratified by age and adjusted potential confounders *.

	45–54 Years Old (*n* = 6714)			55–64 Years Old (*n* = 8560)			65–74 Years Old (*n* = 6123)			75+ Years Old (*n* = 3930)		
	OR	[95% CI]	*p*	OR	[95% CI]	*p*	OR	[95% CI]	*p*	OR	[95% CI]	*p*
*Main model*												
Experienced ACE	1.02	[0.98–1.06]	0.364	1.02	[0.98–1.05]	0.344	0.98	[0.95–1.02]	0.388	0.99	[0.94–1.05]	0.770
*Sensitivity analysis:*												
No ACE	REF		0.785	REF		0.873	REF		0.783	REF		0.353
One ACE	1.02	[0.87–1.21]	0.780	0.96	[0.84–1.09]	0.513	0.98	[0.86–1.11]	0.706	1.08	[0.93–1.25]	0.321
Two ACEs	0.93	[0.77–1.12]	0.430	1.02	[0.88–1.18]	0.811	1.07	[0.91–1.26]	0.434	1.06	[0.87–1.29]	0.565
Three or more ACEs	0.97	[0.81–1.17]	0.764	0.99	[0.85–1.15]	0.869	0.99	[0.84–1.18]	0.936	0.87	[0.69–1.10]	0.251

* Adjusted for age, sex, level of education, ethnicity, and household income. Abbreviations: ACE: adverse childhood experience; OR: odds ratio; CI: confidence interval; REF = reference category.

## Data Availability

The data are available from the CLSA (Available online: www.clsa-elcv.ca (accessed on 11 March 2022)) for researchers who meet the criteria for access to deidentified CLSA data.
